# Changes in Perceived Stress and Lifestyle Behaviors in Response to the COVID-19 Pandemic in The Netherlands: An Online Longitudinal Survey Study

**DOI:** 10.3390/ijerph19074375

**Published:** 2022-04-05

**Authors:** Isabel A. L. Slurink, Veerle R. Smaardijk, Willem J. Kop, Nina Kupper, Floortje Mols, Dounya Schoormans, Sabita S. Soedamah-Muthu

**Affiliations:** 1 Center of Research on Psychological Disorders and Somatic Diseases (CORPS), Department of Medical and Clinical Psychology, Tilburg University, 5000 LE Tilburg, The Netherlands; veerlesmaardijk@hotmail.com (V.R.S.); w.j.kop@tilburguniversity.edu (W.J.K.); h.m.kupper@tilburguniversity.edu (N.K.); f.mols@tilburguniversity.edu (F.M.); d.schoormans@tilburguniversity.edu (D.S.); s.s.soedamah@tilburguniversity.edu (S.S.S.-M.); 2 Institute for Food, Nutrition and Health, University of Reading, Reading RG6 6AR, UK

**Keywords:** COVID-19, lifestyle, health behavior, diet quality, physical activity, prospective cohort

## Abstract

The COVID-19 pandemic has substantial implications for physical and mental wellbeing. This study investigated changes, over time, in lifestyle behaviors and perceived stress during the initial phase of the pandemic and associations with COVID-19 symptoms, in the Dutch general population. An online longitudinal survey study was performed with pre-lockdown measurements in February, and subsequently in April and June 2020 (*n* = 259, mean age 59 ± 14 years, 59% women). Self-report questionnaires were used to assess weight, diet quality, physical activity, alcohol intake, and smoking. Perceived stress was measured using the validated perceived stress scale (PSS-10). The presence of COVID-19 symptoms (yes/no) was defined as fever, or >3 of the following symptoms: weakness/tiredness, muscle ache, dry cough, loss of smell/taste, and breathing difficulties. Data were analyzed using linear mixed models, adjusted for age, sex, educational level, marital status and (change in) employment status. Minimal increases over time were observed in alcohol intake (0.6 ± 0.7 to 0.7 ± 1.1 glasses/day, *p* = 0.001) and smoking (9.5 ± 8.7 to 10.9 ± 9.4 cigarettes/day among 10% smokers, *p* = 0.03), but other lifestyle behaviors remained stable. In April 2020, 15% reported COVID-19-related symptoms, and in June 2020, this was 10%. The presence of COVID-19 symptoms was associated with increased perceived stress (*p*_interaction_ = 0.003) and increased alcohol consumption (*p*_interaction_ = 0.03) over time. In conclusion, in this prospective study, COVID-19 symptoms were associated with increases in perceived stress and alcohol consumption. Future research on biopsychosocial determinants and underlying mechanisms of lifestyle changes, as a response to the COVID-19 pandemic, is needed.

## 1. Introduction

The coronavirus disease 2019 (COVID-19) pandemic poses an enormous burden worldwide, with over 6 152 095 deaths as of April 2022 [1]. The World Health Organization (WHO) declared COVID-19 a pandemic in March 2020, when many countries experienced an alarming increase in COVID-19 incidence. National lockdowns were implemented, restricting social interactions, access to shops and recreation facilities [1]. SARS-CoV-2, the virus causing COVID-19, may induce a persevering immune response, with serious consequences, both for physical and mental health, especially in patients with comorbidities, such as autoimmune diseases and cardiovascular diseases [2,3]. The importance of lifestyle behaviors to prevent and control COVID-19 has become apparent, with studies showing associations with obesity, smoking, poor diet and physical inactivity and higher COVID-19 prevalence, severity, and mortality [4,5,6,7,8].

Current evidence on the impact of COVID-19 on lifestyle comprises numerous cross-sectional surveys, assessing perceived behavioral changes during lockdowns. Many studies show that the initial disruption in daily routine led to decreases in physical activity level [9,10], poorer nutrition and overeating [11,12,13], increased alcohol consumption [11,13], and smoking [10,14,15,16,17,18,19,20]. However, improvements in lifestyle behaviors were also reported [15,21,22,23,24,25,26,27] and considerable parts of populations did not report changes [11,28]. Negative behavioral changes may arise from boredom, motivational problems, and negative emotions [16,25,29]. On the contrary, positive changes may be due to more free time to cook healthy meals and exercise due, in part, to limited social gatherings. Further, people could be motivated to improve their health because of fear of higher susceptibility for COVID-19 and more severe symptoms due to poor health [22].

The role of suspected or confirmed COVID-19 on lifestyle behaviors and perceived stress is unknown. This knowledge is crucial, especially because it has become evident that acute symptoms do not resolve in all patients. For some people, symptoms persist weeks to months after infection (long-COVID-19) [30]. Therefore, the aim of the current study is to assess longitudinal changes in lifestyle behaviors and perceived stress in the early phases of the pandemic and differences because of self-indicated COVID-19 or related symptoms. The results of this study may aid in the design and implementation of public health interventions and regulations. We hypothesized that COVID-19-related symptoms would be associated with adverse lifestyle behaviors, as fever, tiredness and dry cough may hamper being physically active, and loss of smell or taste may alter food preferences [31]. Perceived stress may increase because of fear of contracting COVID-19, worrying about relatives, loneliness, and economic uncertainty, possibly overtaking intentions and self-efficacy in regulating lifestyle behaviors [22,32]. We, therefore, assessed whether the associations between COVID-19 and lifestyle behaviors were independent of perceived stress.

## 2. Materials and Methods

### 2.1. Study Design and Participants

We performed a longitudinal survey study in the general Dutch population. The baseline questionnaire was completed at T0 which was between 13 February and 14 March 2020 in the early phase of the COVID-19 pandemic. At this time, COVID-19 was confirmed in Europe. The first Dutch COVID-19 case was identified on 27 February 2020. Details of the general recruitment and sampling methods have been described previously [33]. In short, participants were recruited as part of an annually distributed survey using age- and sex-stratified non-random quota sampling methods. Inclusion criteria were being aged 18–85 years and proficient in Dutch. At inclusion, participants were told that the study would comprise a single measurement occasion only. In response to the COVID-19 pandemic, participants that took part in this measurement were invited to participate in completing the follow-up questionnaires. The first follow-up questionnaire was completed at T1 from 17 April until 11 May 2020 during the peak of the first wave of the pandemic (for more information see https://coronadashboard.government.nl/, accessed on 5 March 2021). In addition to the basic (hygienic) rules, an intelligent lockdown was adopted from 23 March until 1 June 2020. Measures included stay-at-home advice, social distancing, prohibition of gatherings, and closing of restaurants, bars, the cultural sector, and sporting facilities. The second follow-up questionnaire at T2 was completed from 2 to 22 July 2020, when the intelligent lockdown was lifted, and more restrictions were relaxed. Events were allowed and restaurants, bars, the cultural sector, and sporting facilities were open without a limit on the number of visitors.

The baseline and follow-up studies were approved by the Ethics Review Board of the Tilburg School of Social and Behavioural Sciences of Tilburg University (#RP-055) and participants provided informed consent.

The baseline questionnaire was completed by 1831 participants (Figure S1). Of this group, 426 (23%) participants returned a complete questionnaire at T1. Six (0.3%) returned an incomplete questionnaire, 76 (4%) participants declined participation, and 1323 (72%) did not respond or did not complete the questionnaire. At T2, questionnaires were returned by 259/401 (66%) participants, 4 (1%) returned an incomplete questionnaire, 4 (1%) could not be matched with T0 data based on age and sex, 10 (2%) declined, and 149 (35%) did not respond. Therefore, the final sample included 259 participants with measurements at all timepoints T0, T1 and T2. Data on diet quality and physical activity were not collected at T1 in order to reduce participant burden. These data are therefore available in a subsample of 64 and 85 participants respectively. 

### 2.2. Data Collection and Measures

Participants completed online questionnaires via Qualtrics survey software. After de-identification, participants received a unique number to link the baseline with the follow-up questionnaires. 

#### 2.2.1. Lifestyle Behaviors

*Body Mass Index (BMI)*—At baseline, participants were asked to report their height (cm) and weight (kg) and BMI is reported as kg/m^2^. At follow-up, participants were asked to report their weight.

*Diet quality*—Dietary intake was measured at T0 and T2 using a 34-item Food Frequency Questionnaire (FFQ) [34]. Average daily intakes (grams/day) per item were calculated by multiplying the frequency of consumption by Dutch standard portion sizes (https://portie-online.rivm.nl/, accessed on 5 March 2021). Subsequently, the Dutch Healthy Diet (DHD15)-index score was calculated representing adherence to the Dutch Dietary Guidelines, reflecting diet quality [35]. For the current study, the DHD15-index score was based on 12 out of 15 components since salt, separation between solid and liquid fats, and filtered and unfiltered coffee were not collected. The wholegrain scoring was based on the adequacy subcomponent only since the ratio wholegrain/refined grain was not collected. The sum of the 12 components was calculated, resulting in a total score ranging from 0 (no adherence) to 120 points (maximum adherence). Further details on scoring and mean intakes per component are reported in Table S1.

*Physical activity*—Physical activity in the past 7 days was investigated at T0 and T2. Questions assessed the time spent on physical activities including walking or cycling (e.g., for buying groceries, visiting someone or walking the dog), sports or recreational activities (light, moderate or strenuous intensity), and muscle strengthening exercises. Frequency was assessed as never, seldom (1–2 days/week), sometimes (3–4 days/week) and often (5–7 days/week), and the average duration in the categories less than 1 h, 1–2 h, 2–4 h, and more than 4 h per day. Physical activity level was expressed in average time in minutes/day calculated as frequency multiplied by duration. 

*Alcohol and smoking*—At all measurement occasions, participants were asked to report the frequency and number of alcoholic drinks per week. No distinction between types of alcohol was made. Alcohol intake was categorized as 0, >0–10 g a day (1 glass), >10–20 g (2 glasses), or >20 g (>2 glasses) of alcohol per day. Smoking status was assessed as current, former, or never smoker and participants were asked to indicate the number of cigarettes/cigars per day.

#### 2.2.2. Perceived Stress

The validated 10-item Perceived Stress Scale (PSS-10) was used to measure perceived stress levels in the past month [36]. The score was calculated if at least 7 items were answered. PSS-10 scores can range from 0 to 40 and scores ≥14 were used to identify participants with high levels of perceived stress [37]. The 10-item version of this scale has excellent psychometric properties [38]. In our sample, the Cronbach’s α was 0.87.

#### 2.2.3. Symptom-Based and Self-Indicated COVID-19

Presence or absence of COVID-19 caused by SARS-CoV-2 was operationalized as symptom-based and self-indicated COVID-19. No notable variants of SARS-CoV-2 were present during this study in the Netherlands; the Alpha variant emerged after the present study at the end of 2020. The COVID-19 symptoms variable was constructed based on self-reported symptoms. These symptoms were collected by a modified version of the Common Cold Questionnaire (CCQ) [39], supplemented with COVID-19-specific symptoms such as weakness or tiredness, muscle ache, dry cough, loss of smell or taste, and difficulties with breathing. Participants were asked to indicate frequency (not at all, a couple of days, about a week, more than a week) and severity (none, mild, moderate, severe) of experienced symptoms in the past four weeks (T1) or two months (T2). Furthermore, participants were asked if they had fever (temperature of ≥38 °C) in the past 6 (T1) or 8 weeks (T2). It was previously reported that symptoms are predictive of COVID-19 infectiousness and the main rationale for COVID-19 testing [40]. The COVID-19 symptoms variable was coded as having a fever as this is the most characteristic symptom of COVID-19, or presence of at least three of the following symptoms mildly, moderately, or severely and at least more than a few days: weakness or tiredness, muscle ache, dry cough, loss of smell or taste, and difficulties with breathing (Table 1) [40]. The self-indicated COVID-19 variable was assessed by asking participants if they thought they had COVID-19 (yes/no) (Table 1). Both the COVID-19 symptoms and self-indicated COVID-19 exposures were analyzed separately because the Spearman correlation coefficient between these two variables was moderate, with r = 0.41 at T1 and r = 0.26 at T2.

#### 2.2.4. Sociodemographic Measures

Information on sociodemographic characteristics was collected at baseline and included age, sex, education level, marital status, and employment status. At follow-up, participants were asked to indicate whether their employment status had changed due to the COVID-19 pandemic. The responses ‘I continued to work, but worked from home, I worked less hours’ and ‘my work came to an end’ were categorized as ‘change’. 

### 2.3. Statistical Analysis

Data are reported as mean and standard deviations (SD) for continuous variables, and % (n) for categorical variables. Repeated measures MANOVA for continuous variables and χ2-tests for categorical values were used to assess whether lifestyle behaviors and perceived stress changed from T0 to T1 and T2. 

Random intercept linear mixed models were constructed to examine longitudinal changes and associations of COVID-19 symptoms or self-indicated COVID-19 (yes/no) with perceived stress, BMI, alcohol intake and smoking behavior and lifestyle outcomes. This procedure calculates beta coefficients with 95% confidence intervals, account for missing data and within-subject correlation [41]. Participants with data for lifestyle outcomes available at least at two time points were included in analyses (Figure S1). Confounding variables with missing values at all three time points (sociodemographic characteristics; 5.4%; *n* = 14) were imputed using multiple imputation (*n* = 10). The crude models included the independent variables time (continuous) reflecting longitudinal changes and COVID-19 exposures as fixed effects. The second model included adjustments for sociodemographic characteristics; age, sex, educational level, marital status, employment status and change in employment status. Linearity of time was assumed in all models as quadratic time effects were non-significant. The within-subject effects of COVID-19 on the course of perceived stress, BMI, alcohol intake and smoking behavior were examined by including interactions between time and the COVID-19 exposures. To examine whether associations were independent of perceived stress, we constructed a third model additionally adjusting for perceived stress. A *p*-value ≤ 0.05 (two-sided) was considered statistically significant. All statistical analyses were performed using SPSS (version 24) and R version 3.6.3.

## 3. Results

### 3.1. Participant Characteristics

The mean age was 56.6 ± 14.3 years (range 20–80), 59% were female and 47% had a low or medium educational level (Table 2). More than half of the sample was employed and 81% had a partner. In April 2020 (T1), 40% indicated that their work status had changed. Participants included in the study (*n* = 259) compared to participants who dropped out (*n* = 1572) were older (57 ± 14 vs. 46 ± 16, *p* < 0.001), more often unemployed (41% vs. 25%, *p* = < 0.001), more often former smokers (27% vs. 17%, *p* < 0.001) and had less often moderate or high perceived stress levels (30% vs. 35%, *p* = 0.04) (Table S2).

### 3.2. Changes in Lifestyle Behaviors and Perceived Stress

Table 3 displays the changes in lifestyle and perceived stress levels. A minimal change in daily alcohol intake was observed (0.6 ± 0.7 glasses/day at T0 to 0.7 ± 1.1 glasses/day at T2), yet the percentage of participants consuming more than 1 glass/day increased from 16% at T0 to 23% at T1 and 26% at T2. Furthermore, the percentage of participants consuming no alcohol increased from 14% at T0 to 29% at T1 and 25% at T2. Among smokers (10% of the sample), the number of cigarettes/day increased from 9.5 ± 8.7 at T0 to 10.9 ± 9.4 at T2. In linear mixed models, these increases were significant after adjustments for sociodemographic characteristics, for both alcohol intake (B_glasses/day_: 0.07, 95%CI: 0.04; 0.11, *p* = 0.0001) and smoking (B_cigarettes/day_: 0.68, 95%CI 0.09; 1.28, *p* = 0.03) (Table 4). Factors that did not change over time were perceived stress (~10 ± 7, score range 0–37), BMI (~26.0 ± 4.3), diet quality (~82 ± 16) and physical activity level (~115 ± 104 min/day). Time effects of all lifestyle variables remained similar after additional adjustment for perceived stress.

### 3.3. Association between COVID-19, Lifestyle Behaviors and Perceived Stress

At T1 (April 2020), 15% (*n* = 38/259) reported COVID-19-related symptoms and at T2 (June), this percentage decreased to 10%. When examining participants’ self-indicated presence vs. absence of COVID-19, 21% (*n* = 53/251) thought they had had COVID-19 at T1, which overlapped with having COVID-19 symptoms in 22 participants (9%). At T2, 16% (*n* = 41/257) thought they had had COVID-19, and this corresponded to having COVID-19 symptoms in 12 participants (5%). Notably, 58% of participants with either symptoms or self-indicated COVID-19 at T1 also reported COVID-19 exposure at T2. Five persons were tested for COVID-19, of whom none tested positive. 

The presence of COVID-19-related symptoms and self-indicated COVID-19 were not associated with BMI, diet quality, physical activity, and smoking. The presence of COVID-19-related symptoms was significantly associated with higher perceived stress (B = 1.35, 95%CI: 0.06; 2.64, *p* = 0.04). This association was similar although no longer significant after adjustment for sociodemographic variables (B = 1.26, 95%CI: −0.03; 2.55, *p* = 0.05) (Table 4). 

**Figure 1:**
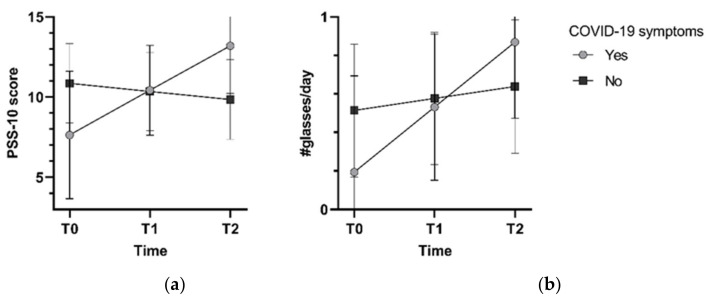
Estimated marginal means for perceived stress (**a**) and alcohol intake (**b**) from models with an interaction of time and COVID-19 symptoms, adjusted for sociodemographic factors.

Furthermore, a significant interaction between time and COVID-19 symptoms was present in models for perceived stress (*p*_interaction_ = 0.003) and alcohol intake (*p*_interaction_ = 0.03) (Figure 1). Perceived stress increased over time in participants with COVID-19 symptoms (estimated marginal mean T0: 7.5, 95%CI 3.52; 11.44 to T2: 13.06, 10.08; 16.04), but not in those without symptoms (~10). Likewise, alcohol intake increased in participants with COVID-19 symptoms (0.18, −0.31; 0.68 to 0.86, 0.46; 1.25 glasses/day), compared to those without COVID-19 symptoms (0.5 to 0.6 glasses/day). Additional adjustment for perceived stress did not change the associations between the COVID-19 exposures and the lifestyle behaviors.

## 4. Discussion

We investigated longitudinal changes in lifestyle behaviors and their associations with COVID-19 in the first four months of the pandemic, in 259 middle-aged adults (aged 56.6 ± 14.3 years) living in the Netherlands. Overall, alcohol intake and smoking increased slightly from February to June 2020, but perceived stress, BMI, diet quality and physical activity remained stable. A unique aspect of this study is that we assessed the impact of two COVID-19 exposures (symptoms and self-indicated COVID-19) on lifestyle behavior, in addition to individuals’ behavioral responses during the pandemic. People with self-reported COVID-19 symptoms showed an increase in perceived stress levels and alcohol intake, whereas people without symptoms had stable perceived stress levels and alcohol intake, independent of sociodemographic characteristics.

The present results on higher perceived stress levels as a consequence of COVID-19 symptoms were in accordance with two cross-sectional studies, conducted at the start of the COVID-19 outbreak in China (*n* = 1210) and Saudi Arabia (*n* = 1669), showing that participants experiencing difficulties with breathing, muscle pain, dizziness and/or headache reported higher perceived stress levels [42,43]. Multiple factors may explain higher perceived stress levels, including fear of worsening symptoms, fear of infecting others, and mandatory self-quarantine measures, resulting in changes in work status and social isolation [44]. Especially in the early phases of the pandemic, uncertainty prevailed concerning the course and severity of infection, cultivated by contradictory information in the media and science [45]. Follow-up studies on the long-term mental health impacts of the pandemic and preventive strategies are needed.

Smoking behavior increased during the pandemic, independent of demographic factors. This finding is consistent with cross-sectional evidence, indicating that the behavioral responses of smokers to COVID-19 are mixed [46,47], yet more smokers seem to have increased their smoking behavior [14,15,16,17,28]. For some, perceived risk of COVID-19 may be a motivator to quit [17], and less social contacts urged less smoking [16], whereas for others, stress, boredom, and maladaptive coping increased smoking [14]. The minimal increase in smoking behavior observed in our study was similar after additional adjustment for (changes in) perceived stress. In contrast to our results, smokers with moderate perceived stress were more likely to either increase or reduce smoking, and smokers with high perceived stress were even more likely to change their smoking habits, in a representative Dutch sample (*n* = 957) in May 2020 [16]. 

In our study, alcohol intake increased marginally over the first four months of the pandemic, especially among people with self-reported COVID-19 symptoms. A recent review showed that mental health was the most reported risk factor in increased alcohol use during the pandemic [48]. In the Netherlands, the strictness of regulations, including closing restaurants and bars, the curfew and the alcohol ban in supermarkets after 8pm, did not affect the percentage of people changing their alcohol intake [49]. In the long term, both increases and decreases in alcohol intake can be expected due to distress related to the pandemic [48,50], and further research is needed. 

We did not find changes over four months of follow-up or associations between COVID-19, body weight, diet quality and physical activity. This is in accordance with two other Dutch studies: a cross-sectional survey (*n* = 1030), showing that most people (83%) reported no weight change [51], and the Lifelines COVID-19 cohort (*n* > 70,000), showing no changes in BMI (between 26.1–26.2 kg/m^2^) from March 2020 to May 2021 [52]. On the contrary, a systematic review of 36 international observational studies in the first lockdown period showed that body weight increased in 11.1–72.5% of individuals and decreased in 7.2–51.4% [53]. The lockdown measures in the Netherlands were less strict compared to, for example, Southern European countries or China, possibly explaining the absence of major changes in body weight in our study [21,23]. Diet quality might not have changed, as it does not include snack consumption and overeating, which did increase during the pandemic in several studies [11,12,13]. The overall stable physical activity level in our study was also in contrast with previous literature showing decreased physical activity levels during the early phases of the pandemic [21,22]. Possibly because the first measurement took place in winter, and the last in summer, the difference in season might explain this inconsistency [23]. Further, sporting facilities reopened, and outdoor group sports were allowed again in June.

Furthermore, our results may be explained by the characteristics of included participants, who were somewhat older, more often unemployed, and reported less perceived stress compared to participants who dropped out. Younger populations might be more prone to adverse lifestyle changes during the pandemic, due to adverse psychological symptoms caused by caregiving roles, changes in job status and financial issues [45]. Indeed, a French study (*n* = 20,557) showed that older age, not working and less anxiety and/or fewer depressive symptoms were associated with no lifestyle changes [25]. Characteristics associated with unfavorable changes included younger age, female sex, non-smoker, high educational level, working from home, having children and higher levels of anxiety and/or depressive symptoms. With larger samples, profile analysis of longitudinal trajectories could unveil determinants of change patterns, defining vulnerable subpopulations and aiding targeted interventions.

Similar to our study, no association between COVID-19 (including relatives/friends with COVID-19) and food intake was found in another Italian sample (*n* = 490); however, COVID-19 was associated with both increases and decreases in physical activity [15]. Furthermore, a cross-sectional study in Scotland (*n* = 399) reported no association between suspected or confirmed COVID-19 and physical activity but found a possible association between living with someone with suspected/confirmed COVID-19 and having higher physical activity levels [19]. In Dutch and Belgian patients with confirmed COVID-19 and persistent symptoms (*n* = 239), participants reported that they spent less time on walking and sports at three months of follow-up compared to before symptom onset [54]. These associations warrant further exploration, as increases in sedentary time due to COVID-19 are negatively correlated with global mental health, depression, anxiety, and quality of life, irrespective of age [10].

### Strengths and Limitations

A major strength of the present study is the longitudinal assessment of lifestyle behaviors. This enabled quantitative assessments of change in lifestyle behaviors, limiting response bias in socially desirable behavior, as opposed to retrospective measures of perceived change [55]. There are also limitations that we need to address. First, there was a large drop-out rate in this study (86%), possibly as at inclusion, participants were told that the study would comprise one measurement occasion only. The response rate for diet quality and physical activity were substantially lower, as the original study focused on mental health; therefore, questionnaires of diet quality and physical activity were not included at T1 due to participant burden with long questionnaires. Participants included in the study were somewhat older, more often unemployed, and reported less perceived stress compared to participants that did not take part in follow-up measurements. Our final sample was more often more highly educated compared to the general Dutch population (53% vs. 41%), was less often employed (59% vs. 70%), reported less current smoking (10% vs 14.9%), but was comparable regarding overweight/obesity (53% vs. 50% with a BMI ≥ 25 kg/m^2^) [25,26]. Thus, the results should be interpreted in light of possible selection bias, and generalizability might be limited. Second, we present estimates adjusted for sociodemographic confounders; nevertheless, residual confounding is possible and inherent to the observational design. Third, changes over time might, to some extent, be explained by regression to the mean, plausibly due to a ceiling effect, as participants with extreme values at baseline can hardly score higher or lower than this extreme value at follow-up [15]. Furthermore, measurement errors might cause regression to the mean. Nevertheless, the observed increases in smoking behavior and alcohol intake in our study agreed with previous literature. Lastly, the questionnaires were based on self-report, which are prone to response bias. The COVID-19 outcomes could not be verified by diagnostic test results, as PCR tests in case of symptoms only became available after our study, from 1 June 2020 onwards, and testing capacity was low. We tried to minimize this response bias by focusing merely on the presence of COVID-19-related symptoms, not on severity or duration, with the assumption that people can accurately identify the presence of weakness or tiredness, muscle ache, dry cough, loss of smell or tase or difficulty breathing. The self-indicated COVID-19 outcome relies on the ability of people to determine if they were infected, justified as possible symptoms of COVID-19 were widely communicated by health organizations and workplaces. Nevertheless, asymptomatic infections may not have been completely captured in our study. Supposedly, people with an asymptomatic infection do not seek diagnostic testing and are less likely to change their lifestyle behavior, as compared with people who have a notable COVID-19 infection. Verification of COVID-19 outcomes with diagnostic testing in future studies is warranted.

## 5. Conclusions

In conclusion, alcohol intake and smoking increased slightly from February to June 2020. Participants with (vs. those without) COVID-19 symptoms reported increased levels of perceived stress and alcohol intake. This underlines the need to increase our understanding of the psychological needs of individuals presenting with symptoms, effectuating sufficient testing capacity and healthcare for patients with a less severe course of infection. COVID-19 symptoms and self-indicated COVID-19 were not associated with BMI, diet quality, physical activity, and smoking. Future studies could further investigate explanations for changes in lifestyle behaviors, as a response to both short-term symptoms and long-COVID-19, with longer follow-up, and subgroup differences, taking a biopsychosocial approach to COVID-19 prevention and treatment [56]. Efforts need to be made to engage people in a healthy lifestyle, to limit the burden on our healthcare systems, avoid further social and behavioral restrictions, and promote health beyond the context of the pandemic.

## Figures and Tables

**Table 1:** COVID-19 indicator coding based on questionnaire responses of the study sample (*n* = 259) in April 2020 (T1) and June 2020 (T2).

Indicator	Coding	Questionnaire Responses	T1, *n* (%)	T2, *n* (%)
COVID-19 symptoms	Yes	Fever AND/OR	13 (5%)	8 (3%)
		At least 3 of following symptoms:		
		Weakness of tiredness	91 (36%)	78 (30%)
		Muscle ache	50 (20%)	35 (14%)
		Dry cough	61 (24%)	30 (12%)
		Loss of smell or taste	18 (7%)	13 (5%)
		Difficulty breathing	26 (10%)	21 (8%)
Self-indicated COVID-19	Yes	Yes, I think that I have been infected, but I had no symptoms	17 (7%)	12 (5%)
		Yes, I had symptoms, but no fever	25 (10%)	17 (6%)
		Yes, I had symptoms and fever	7 (3%)	11 (4%)
		Yes, I contacted my general practitioner because of my symptoms	7 (3%)	2 (1%)
		Yes, I was tested for Coronavirus	0 (0%)	0 (0%)
	No	No	202 (78%)	216 (82%)
		Yes, but I tested for Coronavirus, and the test was negative	0 (0%)	5 (2%)

**Table 2:** Sociodemographic characteristics of the study sample (*n* = 259) ^1^.

Demographic Factors	
Age (years)	56.6 ± 14.3
Female sex	157 (59%)
With partner ^2^	206 (81%)
Low/medium educational level ^3^	121 (47%)
Employment status	
Working full-time	60 (23%)
Working part-time	92 (36%)
Other ^4^	104 (41%)
Change in employment status, April 2020	
No change	57 (23%)
Change	101 (40%)
Other/not applicable ^4^	94 (37%)

^1^ Values are mean ± SD for continuous variables or n, % for categorical variables. ^2^ With partner: married/registered partners, living together with partner, not living together with partner. Without partner: single, divorced, widow/widower. ^3^ Low: primary education, secondary education, or secondary vocational education, high: university education or higher professional education. ^4^ Other employment status including e.g., retired, studying, unemployed, homemaker, disability pension.

**Table 3:** Perceived stress and lifestyle behaviors of the study sample (*n* = 259) according to measurement moment: January 2020 (T0), April 2020 (T1) and June 2020 (T2) ^1^.

	T0	T1	T2	*p*-Value ^2^
**Perceived stress**	*n* = 258	*n* = 256	*n* = 247	
Perceived stress score (PSS-10)	10.5 ± 6.9	9.7 ± 6.2	9.8 ± 6.6	0.05
Moderate or high (PSS-10 ≥ 14)	76 (30%)	66 (26%)	60 (24%)	0.40
**Lifestyle behaviors**				
*Weight*	*n* = 258	*n* = 249	*n* = 246	
BMI (kg/m^2^)	25.9 ± 4.3	26.0 ± 4.3	26.0 ± 4.3	0.15
Overweight/obese (BMI ≥ 25 kg/m^2^)	135 (53%)	137 (56%)	(137) 56%	0.69
*Diet quality*	*n* = 78		*n* = 186	
DHD15-index score	81.8 ± 16.3		81.6 ± 14.9	0.54
Range	41–116		40–112	
*Physical activity level*	*n* = 92		*n* = 240	
Minutes/day	119 ± 104		115 ± 105	0.53
Range	0–439		0–778	
*Smoking status*	*n* = 257	*n* = 250	*n* = 245	
Never	163 (63%)	157 62%)	154 (62%)	0.99
In the past	70 (27%)	73 (29%)	72 (29%)	
Current	27 (10%)	24 (9%)	24 (10%)	
# cigarettes/day among smokers (*n* = 32)	12.0 ± 8.1	11.9 ± 8.3	13.1 ± 8.8	0.03
*Alcohol intake*	*n* = 259	*n* = 243	*n* = 239	
# glasses/day	0.6 ± 0.7	0.6 ± 0.8	0.7 ± 1.1	<0.001
Alcohol intake category				<0.001
0 g/d	36 (14%)	74 (29%)	64 (25%)	
>0 to 10 g/d (<1 glass/d)	182 (70%)	107 41%)	104 (40%)	
>10 to 20 g/d (1–2 glasses/d)	22 (9%)	41 (16%)	43 (17%)	
>20 g/d (>2 glasses/d)	19 (7%)	17 (7%)	22 (9%)	

^1^ Values are mean ± SD for continuous variables or n, % for categorical variables. ^2^ *p*-values from χ^2^ for categorical values or repeated measures MANOVA for differences between T0, T1 and T2. DHD15, Dutch Healthy Diet index 2015; PSS-10, Perceived Stress Score.

**Table 4:** Regression coefficients (unstandardized betas with 95% confidence intervals) of time effects and associations between presence of COVID-19-related symptoms or self-indicated COVID-19, and perceived stress, BMI, diet quality, physical activity, smoking and alcohol intake during the COVID-19 epidemic ^1^.

	Time		COVID-19 Symptoms		COVID-19 Self-Indicated	
	B (95% CI)	*p-Value*	B (95% CI)	*p-Value*	B (95% CI)	*p-Value*
*Stress (PSS-10) score*						
Model 1	−0.34 (−0.68; 0.003)	0.05	**1.35 (0.06; 2.64)**	**0.04**	0.40 (−0.78; 1.57)	0.51
Model 2	−0.33 (−0.67; 0.01)	0.06	1.26 (−0.03; 2.55)	0.05	0.19 (−0.98; 1.36)	0.75
*BMI (kg/m^2^)*						
Model 1	0.06 (−0.02; 0.13)	0.15	0.03 (−0.27; 0.33)	0.84	−0.16 (−0.44; 0.11)	0.24
Model 2	0.06 (−0.02; 0.13)	0.15	0.03 (−0.27; 0.33)	0.84	−0.16 (−0.44; 0.11)	0.25
Model 3	0.05 (−0.02; 0.13)	0.16	0.04 (−0.26; 0.34)	0.78	−0.15 (−0.42; 0.13)	0.29
*Diet quality (DHD15-index score)*						
Model 1	−0.66 (−2.07; 0.76)	0.36	0.03 (−7.23; 7.28)	0.99	0.78 (−5.76; 7.33)	0.81
Model 2	−0.66 (−2.07; 0.76)	0.36	0.92 (−6.24; 8.07)	0.80	0.12 (−6.42; 6.65)	0.97
Model 3	−0.82 (−2.22; 0.58)	0.25	0.33 (−6.82; 7.49)	0.93	0.36 (−6.09; 6.81)	0.91
*Physical activity (minutes/day)*						
Model 1	−4.75 (−14.97; 5.48)	0.36	5.62 (−46.89; 58.14)	0.83	−6.22 (−52.50; 40.06)	0.79
Model 2	−4.75 (−14.97; 5.48)	0.36	4.71 (−47.19; 56.61)	0.86	−2.68 (−49.15; 43.79)	0.91
Model 3	−5.24 (−15.55; 5.06)	0.32	3.67 (−49.47; 56.81)	0.89	−1.82 (−48.44; 44.80)	0.94
*Smoking (# cigarettes/day)*						
Model 1	**0.68 (0.09; 1.28)**	**0.03**	−0.88 (−2.59; 0.84)	0.31	−1.58 (−3.58; 0.42)	0.12
Model 2	**0.68 (0.09; 1.28)**	**0.03**	−0.85 (−2.57; 0.86)	0.33	−1.56 (−3.56; 0.45)	0.13
Model 3	**0.72 (0.10; 1.33)**	**0.02**	−0.88 (−2.62; 0.86)	0.32	−1.76 (−3.88; 0.36)	0.10
*Alcohol intake (# glasses/day)*						
Model 1	**0.07 (0.04; 0.11)**	**0.0001**	0.05 (−0.10; 0.19)	0.55	0.02 (−0.11; 0.15)	0.74
Model 2	**0.07 (0.04; 0.11)**	**0.0001**	0.06 (−0.09; 0.21)	0.45	0.03 (−0.10; 0.16)	0.62
Model 3	**0.08 (0.04; 0.11)**	**0.0001**	0.05 (−0.10; 0.20)	0.50	0.04 (−0.09; 0.17)	0.58

^1^ Stress, BMI and alcohol intake in 259 participants, COVID-19 symptoms, T1: *n* = 38 (15%), T2: *n* = 26 (10%), COVID-19 self-indicated, T1: *n* = 53 (21%), T2: *n* = 41 (16%). Diet quality in 64 participants, COVID-19 symptoms, T1: *n* = 9 (14%), T2: *n* = 10 (16%), COVID-19 self-indicated, T1: *n* = 18 (28%), T2: *n* = 13 (20%). Physical activity in 85 participants, COVID-19 symptoms, T1: *n* = 11 (13%), T2: *n* = 11 (13%), COVID-19 self-indicated, T1: *n* = 22 (26%), T2: *n* = 15 (18%). Smoking in 32 smokers (at any time point), COVID-19 symptoms, T1: *n* = 9 (28%), T2: *n* = 6 (19%), COVID-19 self-indicated, T1: *n* = 8 (25%), T2: *n* = 5 (16%). Model 1 included time (continuous) as fixed effect (crude model). Model 2 additionally adjusted for age (continuous), sex (female/male), educational level (low/high), marital status (single/in relationship), employment status (full-time/part-time/other) and change in employment status (no change/change/other). Model 3 additionally adjusted for perceived stress. Significant results indicated in **bold**. COVID-19, coronavirus Disease 2019; DHD15, Dutch Healthy Diet; PSS-10, Perceived Stress Scale.

## Data Availability

Data described in the manuscript, code book, and analytic code will be made available upon reasonable request, pending approval by the authors.

## Supplementary Materials

The following supporting information can be downloaded at: https://www.mdpi.com/article/10.3390/ijerph19074375/s1, Figure S1. Flow-chart of study participants; Table S1. Daily intake of food group included in the DHD15-index score; Table S2. Comparison of sample included in the analysis and participants who did not take part in the follow-up analyses.
