# Supplementary material for: Changes in Perceived Stress and Lifestyle Behaviors in Response to the COVID-19 Pandemic in The Netherlands: An Online Longitudinal Survey Study
Source: Int J Environ Res Public Health. 2022 Apr 5;19(7):4375. doi: 10.3390/ijerph19074375 (PMC8998601; doi:10.3390/ijerph19074375)
Supplement: Supplementary file 1 [file ijerph-19-04375-s001.zip › Online_supplemental_figuretables.pdf]

Supplemental files

Changes in lifestyle behaviors and perceived stress in response to the COVID-19 pandemic: a longitudinal survey study

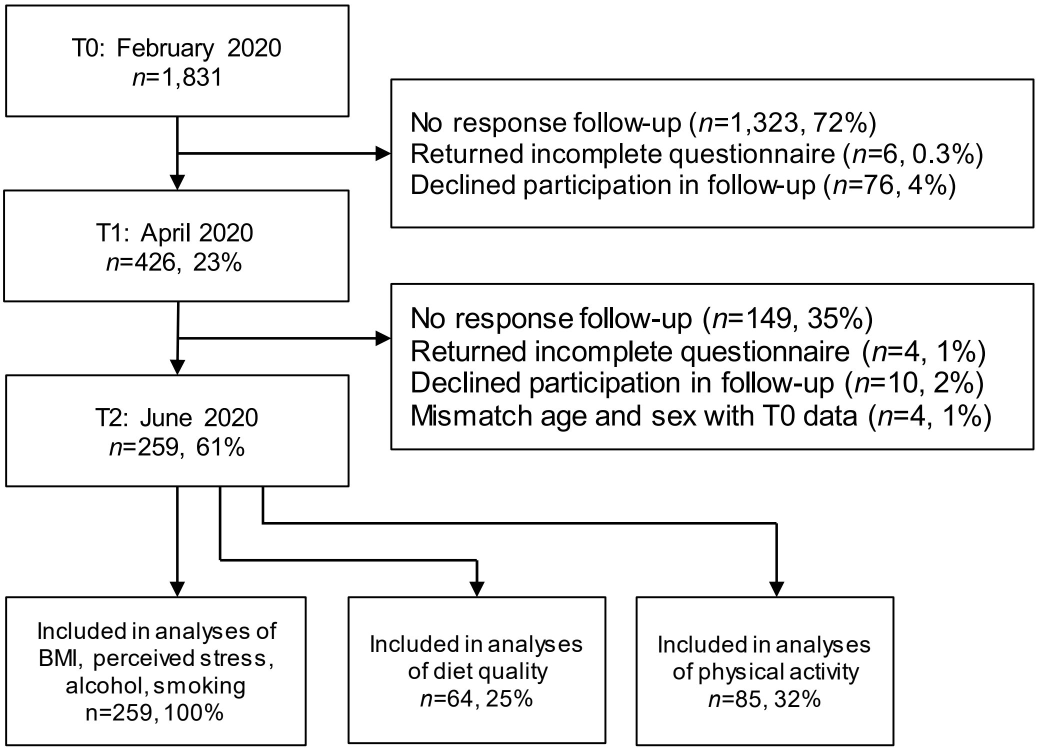

Figure S1. Flow-chart of study participants.

Table S1. Daily intake of food group included in the DHD15-index score.[1]

	**T0 (n=95)**	**T2 (n=238)**	**P-value[2]**
DHD15-index components:	Grams/day, mean ± SD	Grams/day, mean ± SD	
Fruit	132 ± 79	140 ± 86	.15
Vegetables	138 ± 51	155 ± 143	.20
Wholegrain	103 ± 81	95 ± 77	.75
Legumes	17 ± 19	22 ± 38	**.002**
Nuts	11 ± 13	10 ± 18	.47
Dairy	276 ± 159	290 ± 202	.73
Tea	250 ± 229	206 ± 229	.03
Red meat	21 ± 23	23 ± 26	.97
Processed meat	16 ± 23	15 ± 21	.47
Sugar sweetened beverages	19 ± 21	19 ± 17	.77
Fish	131 ± 222	132 ± 202	.85
Alcohol [ethanol]	6 ± 7	7 ± 11	**.005**

[1] The intake of salt, separation between solid and liquid fats, and filtered and unfiltered coffee were not collected. Adequacy components include vegetables, fruit, legumes, nuts, fish, wholegrains and tea, moderation components include red meat, processed meat, sweetened beverages and fruit juices, and alcohol and optimum components include dairy.

[2] P-values from repeated measures MANOVA for differences between T0 and T2.

DHD15, Dutch Healthy Diet.

Table S2. Comparison of sample included in the analysis and participants who dropped out.[1]

	**Participants included** (*n*=259)	**Participants who dropped out** (*n*=1,572)	***p*-value**[2]
Demographic factors			
Age (years)	57 ± 14	46 ± 16	<.001
Female sex	59% (152)	53% (837)	.11
With partner[3]	81% (206)	78% (1,217)	.44
Low/medium educational level[3]	47% (121)	52% (812)	.13
Employment status			<.001
Working full-time	23% (60)	42% (650)	
Working part-time	36% (92)	33% (513)	
Other[3]	41% (104)	25% (392)	
Perceived stress	11 ± 7	12 ± 6	.01
Moderate or high perceived stress (PSS-10 ≥14)	30% (76)	35% (523)	.04
Lifestyle variables			
Diet quality (DHD15-index score)	82 ± 16	78 ± 16	.10
Physical activity level (minutes/day)	119 ± 104	126 ± 124	.57
Smoking status			<.001
Never	63% (162)	71% (1,111)	
In the past	27% (69)	17% (267)	
Current	10% (26)	12% (188)	
# cigarettes/day among smokers	9 ± 9	9 ± 6	.98
Alcohol intake (# glasses/day)	0.6 ± 0.7	0.6 ± 0.8	.76
Alcohol intake category			.53
0 g/d	14% (36)	12% (181)	
>0 to 10 g/d (<1 glass/d)	70% (182)	70% (1,081)	
>10 to 20 g/d (1-2 glasses/d)	9% (22)	11% (168)	
>20 g/d (>2 glasses/d)	7% (19)	8% (124)	

[1] Values are mean (SD) for continuous variables or % (n) for categorical variables.

[2] Independent sample t-tests were performed for continuous variables, Mann-Whitney U tests for not normally distributed continuous variables, and χ2-tests for categorical variables.

[3] With partner: married/registered partners, living together with partner, not living together with partner. Without partner: single, divorced, widow/widower. Low: primary education, secondary education, or secondary vocational education, high: university education or higher professional education. Other employment status including e.g., retired, studying, unemployed, homemaker, disability pension.

DHD15, Dutch Healthy Diet; PSS-10, Perceived Stress Scale.
